# Synthesis and Characterization of Renewable Polyester Coil Coatings from Biomass-Derived Isosorbide, FDCA, 1,5-Pentanediol, Succinic Acid, and 1,3-Propanediol

**DOI:** 10.3390/polym10060600

**Published:** 2018-05-29

**Authors:** Mónica Lomelí-Rodríguez, José Raúl Corpas-Martínez, Susan Willis, Robert Mulholland, Jose Antonio Lopez-Sanchez

**Affiliations:** 1 Stephenson Institute for Renewable Energy, Department of Chemistry, University of Liverpool, Crown Street, Liverpool L69 7ZD, UK; Jose.Raul.Corpas-Martinez@liverpool.ac.uk; 2 Becker Industrial Coatings Ltd, Goodlass Road, Speke, Liverpool L24 9HJ, UK; susan.willis@beckers-group.com (S.W.); Robert.Mulholland@beckers-group.com (R.M.)

**Keywords:** biomass, coatings, isosorbide, FDCA, polyester, biopolymer, 1-5-pentanediol

## Abstract

Biomass-derived polyester coatings for coil applications have been successfully developed and characterized. The coatings were constituted by carbohydrate-derived monomers, namely 2,5-furan dicarboxylic acid, isosorbide, succinic acid, 1,3-propanediol, and 1,5-pentanediol, the latter having previously been used as a plasticizer rather than a structural building unit. The effect of isosorbide on the coatings is widely studied. The inclusion of these monomers diversified the mechanical properties of the coatings, and showed an improved performance against common petrochemical derived coatings. This research study provides a range of fully bio-derived polyester coil coatings with tunable properties of industrial interest, highlighting the importance of renewable polymers towards a successful bioeconomy.

## 1. Introduction

**Figure 1:**
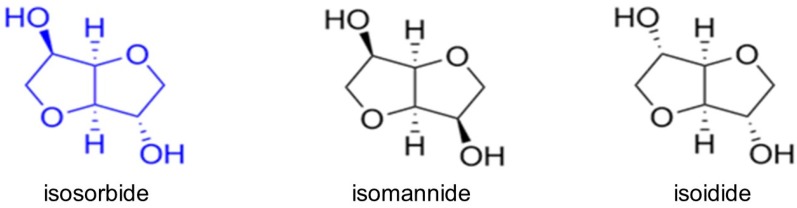
Molecular structures of isosorbide, isomannide, and isoidide.

The environmental and sustainability problems currently faced worldwide call for the implementation and usage of renewable materials across industries. Within polymers, the future in polyesters relies on the inclusion of monomers sourced from bioderived feedstocks [1]. In this vein, our group has previously developed the synthesis, kinetic modelling, and process optimization and intensification of bioderived polyesters based on furan 2,5-dicarboxylic acid (FDCA), succinic acid, 1,3-propanediol, and 1,5-pentanediol [2,3,4]. Common polyester coatings are normally prepared from diacids such as terephthalic acid, isophthalic acid, phthalic anhydride, and adipic acid. The polyalcohols could include difunctional monomers such as neopentyl alcohol, ethylene glycol, or polyfunctional compounds, including trimethylol propane, among other compounds exceeding two functionalities [5]. In this regard, carbohydrates are a vast source of renewable, biomass-derived diols suitable for coil coatings, such as 1,4:3,6-dianhydrohexitols [6]. Dianhydrohexitols are a by-product of the starch industry obtained by the reduction of hexose sugars followed by dehydration. 1,4:3,6-dianhydro-D-glucitol (isosorbide), 1,4:3,6-dianhydrohexitol-D-mannitol (isomannide), and 1,4:3,6-dianhydro-L-iditol (isoidide) are the three main diastereoisomers derived from D-glucose, D-mannose, and L-fructose [7], respectively, and are shown in Figure 1. The reader is encouraged to refer to additional sources for detailed information on the synthesis, chemistry, and properties of 1,4:3,6-dianhydrohexitols [8,9,10]. Isosorbide bears a considerable potential for the production of new tailored chemicals from renewable resources as it is conformed by two *cis*-connected tetrahydrofuran rings with secondary hydroxyl groups in the 2- (*endo*) and 5- (*exo*) positions, which allow for further functionalization or direct processing [9]. The use of isosorbide in polyesters can be motivated by several features: rigidity, chirality, non-toxicity [8], and recently, its use as a monomer for the preparation of UV-cured coatings has been highlighted [11]. Isosorbide has a relatively high thermostability and low segmental mobility, and can be used to improve the glass transition temperatures of polyesters [12]. However, the hydroxyl group in *endo* position easily forms intra-molecular hydrogen bonds with the oxygen in the other ring, which leads to the poor reactivity of the secondary hydroxyl group and the low number average molecular weights of copolyesters [13]. 

Previous work on isosorbide-based polyesters has been reported, mainly based on a variety of biomass-derived monomers, mainly citric acid [14], lactic acid [15], succinic acid [16,17,18,19], sebacic acid [17,20], itaconic acid [18], 1,4-cyclohexanedimethanol [21], and dimethyl-2,5-furan dicarboxylate [21,22]. Goerz et al. [18] studied the synthesis of polyesters from isosorbide, itaconic acid and succinic acid. The obtained polyesters had glass transition temperatures (T_g_) from 57 °C to 65 °C and molecular weights from 1200 Da up to 3500 Da, depending on the molar ratio of the monomers. In the field of coatings, Noordover et al. [14,19,23] reported the synthesis of terpolyesters for powder coatings based on isosorbide, succinic acid, citric acid, and aliphatic diols such as 1,3-propanediol, 1,4-butanediol, and neopentyl glycol, showing number average molecular weights (M_n_) from 2700 up to 4600 Da, and highlighting the effect of isosorbide content on the glass transition temperature [19]. Gioia et al. [24] synthesized polyesters based on recycled PET, succinic acid, and isosorbide for powder coating applications

Jacquel et al. [25] prepared bioderived copolyesters of succinic acid and isosorbide by varying the mol % isosorbide from 5 to 20%. The polyesters had T_g_ from −28 °C to −11 °C, which increased by increasing the mol % isosorbide, while the esterification yield decreased. Zhou et al. [12,13,20] studied the properties and crystallization kinetics of copolyesters based on isosorbide, sebacic acid, and either 1,10-decanediol [20] or 1,3-propanediol [12]. The authors varied the mol % isosorbide from 5.3 mol % to 66.2%, reporting T_g_ ranging from −26 °C to −5 °C, although no glass transition was observed when the mol % isosorbide was below 30%. The M_n_ range was broad, with the polyester of isosorbide and sebacic acid showing the lowest M_n_ (2800 Da), whereas the copolyester with 25.4 mol % isosorbide had the highest M_n_, 17000 Da. No relationship was found between the M_n_ and the amount of incorporated isosorbide [20].

Besides the influence of isosorbide on the final polymer, the effect of catalysts has been studied. For instance, during the synthesis of poly(ethylene terephthalate-co-isosorbide terephthalate), the authors found that combinations of antimony oxide with lithium, magnesium or aluminum based salts successfully increased the efficiency of the transesterification step [26].

Furan 2,5-dicarboxylic acid has been subject to extensive research over the last years, with a drive to develop it as a green chemical building block for polyesters [27,28,29,30,31,32,33]. Furan 2,5-dicarboxylic acid is a versatile, bioderived carboxylic acid which was envisioned as a replacement for terephthalic acid in the synthesis of poly(ethylene terephthalate) (PET) and poly(butylene terephthalate) (PBT), although many other polyfuronoates have been developed. Poly(ethylene 2,5-furandicarboxylate) (PEF) shows greatly improved barrier and mechanical properties, higher glass transition temperature, reduced oxygen permeability, and slower chain mobility than its terephthalic acid counterpart [34]. The reactivity and kinetic modelling of the catalyst effect on the PEF polycondensation reaction was also recently reported [35]. The synthesis of potentially 100% renewable FDCA copolyesters with different biomonomers has been explored as well, namely with succinic acid [2,3,30,33,36,37,38], 1,3-propanediol [2,39], along with some work done with copolyesters of FDCA with lactic acid [40], and isosorbide or its derivatives [21,22,25,41,42]. Tsanaktsis et al. [32] reported for the first time the synthesis of poly(pentylene furonoate) (PPeF) along with poly(heptylene furonoate) (PHepF). Poly(pentylene furonoate) was identified as a semicrystalline polyester with a melting point at 94 °C, T_g_ at 19 °C, and a maximum decomposition temperature at 394 °C. The same research group studied the thermal decomposition mechanism of PPeF, PHepF, and poly(nonylene furanoate) (PNF) [43]. The authors found that the decomposition of PPeF released gases, such as CO and CO_2_, along other degradation products such as dienes, and vinyl- and carboxyl-terminated molecules. 

Lately, the thermal properties of FDCA polyesters have been extensively studied, such as poly(octylene furanoate) [44], along with thermal degradation of different polyfuronoates [45,46]. The potential of polyesters based on FDCA is steadily increasing and their industrialization and commercialization will eventually become a reality. Papageorgiou et al. [47] recently reviewed the current status and latest progress of polyfuranoates.

This research study presents the development and synthesis of new polyester resins based on isosorbide, FDCA, succinic acid, and 1,5-pentanediol to be used in coil coating applications. 1,5-pentanediol has normally been used as a polymerization additive (plasticizer) [48] rather than a main diol during poleysterification; however, we have considered it since its bioderived synthesis is playing a major role within the biorefinery concept, with promising prospects [49,50,51,52,53,54]. Resins with 1,3-propanediol are included as comparison. The paper focuses on the real applicability of the bioderived coatings instead of providing a detailed description on their compositions or chemical characterization. Instead, common mechanical testing analyses for coatings are included. Our objective is therefore to compel a fully biomass-derived, coil coatings library not only of the base polyester resins, but also of the final coatings. We believe our overall work delivers a good basis for the implementation of biomass-derived polymers in large scale and aims to become a strong industrial reference to embrace renewable feedstocks.

## 2. Materials and Methods

### 2.1. Materials

Furan 2,5-dicarboxylic acid (>98%) was purchased from Manchester Organics Ltd. (Runcorn, UK) Isosorbide (>98%), succinic acid (>99%), and 1,5-pentanediol (>99%) were purchased from Acros Organics (England, UK). SnCl_2_ (>98%) was purchased from Alfa Aesar (Haverhill, MA, USA). All other chemicals were of analytical grade and obtained either from Sigma Aldrich (St. Louis, MO, USA) or Fisher Scientific (Hampton, NH, USA).

### 2.2. Synthesis of Isosorbide-Based Renewable Polyesters

**Scheme 1:**
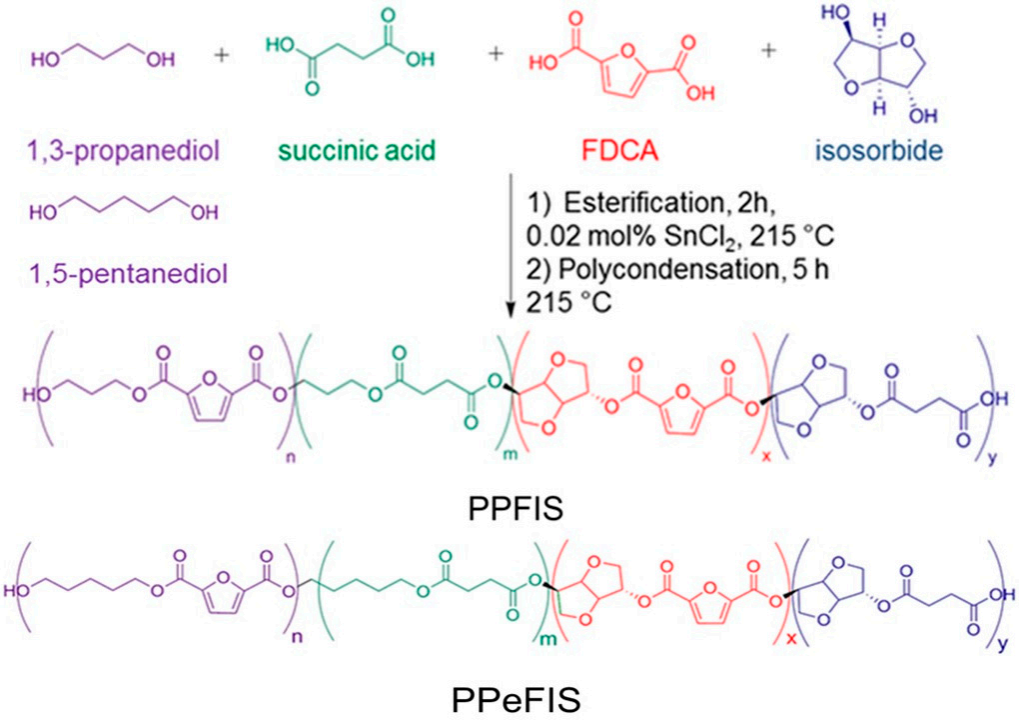
Synthesis of isosorbide-based polyesters with succinic acid, furan 2,5-dicarboxylic acid (FDCA), 1,3-propanediol (PPFIS), and 1,5-pentanediol (PPeFIS).

The structures and the synthetic procedure of the isosorbide polyesters with 1,3-propanediol (PPFIS) and 1,5-pentanediol (PPeFIS) are depicted in Scheme 1, whereas Table 1 and Table 2 summarize the polyesters synthesized, along with their M_n_, weight-average molecular weight (M_w_), and dispersity (Đ), which were calculated by gel permeation chromatography (GPC), following the method described later on in Section 2. The polyesters’ nomenclature is based on the diol used, the diacid molar ratio, and the molar proportion of isosorbide present. For a typical polyester, the first *P* refers to the suffix *poly*, followed either by *P* or *Pe*, if synthesized with either 1,3-propanediol or 1,5-pentanediol, respectively. Next, the notation *F_x_*, indicates the presence of FDCA in *x* mol %. Similarly, *S_y_* refers to the succinic acid present in *y* mol % whilst *I_z_* corresponds to the *z* mol % isosorbide.

Polyesterification reactions were performed at two different scales: 250 mL and 500 mL. The choice of scale depended on the mol % FDCA in the feed, because the bulk viscosity of the system increased as the mol % FDCA was increased. Hence, all the polyesters bearing furanic content above 50 mol % were synthesized at the 500 mL scale. The different scales also facilitated cleaning and recovery of the product, while minimizing the use of solvents. Despite working with two different volumes, the geometry of the stirrer and the shape of the reactor (round-bottom) were the same in both configurations, as well as the nitrogen flow rate. (No flowmeter was in place but the nitrogen flow was set to 2 bubbles·s^−1^). The general polymerization reactors set-up and procedure are available in our previous publications [2,3,4].

The experimental synthesis was followed for all polyesters by adjusting the ratio of the monomers accordingly. The exact quantities for each polyester are available in Supplementary Material Tables S1 and S2, along with the purification polyester method. In a typical polymerization to synthesize PPF_15_I_30_S_85_ to a 250 mL 4-neck-round bottom flask equipped with an overhead stirrer, was added 59 g (0.77 mol) of 1,3-propanediol, 48 g (0.33 mol) of isosorbide, and 17 g (0.11 mol) of FDCA. Secondly, the reactor was heated up to 150 °C and 74 g (0.62 mol) of succinic acid and SnCl_2_ were added. The temperature was increased to 215 °C and was continuously stirred at 350 ppm. The esterification stage was completed after 2 h when all the water had been released and the head temperature on top of the distillation column was back to ambient temperature. Then, the polycondensation reaction was carried out by azeotropic distillation by changing the packed column to a Dean Stark trap, adding 3 wt % xylene as azeotropic agent under atmospheric pressure for 5 hours. The reaction was finally cooled down and the polymer was collected and purified for characterization. The polyesters’ color depended on the amount of FDCA and isosorbide in the mixture. Although SnCl_2_ was the only catalyst used, tin catalysts showed less intense coloration in a comparative polycondensation study using titanium catalysts with furanoates [35]. The intensity color range of the polyesters went from a light yellow to brown with increasing FDCA, although no color space system measurements were undertaken, as our coatings did not require any color specification.

### 2.3. Characterization Methods

#### 2.3.1. Nuclear Magnetic Resonance Spectroscopy (^1^H NMR)

^1^H NMR measurements were performed on a Brucker NMR spectrometer (400 MHz). Deuterated chloroform (CDCl_3_) was used as solvent for all samples.

#### 2.3.2. Gel Permeation Chromatography

Gel permeation chromatography was carried out on an Agilent 1260 Infinity with two Agilent ResiPore Organic 250 × 4.6 mm columns, a guard column, and a refractive index detector. The eluent was tetrahydrofuran (THF) at a flow rate of 0.3 mL/min. Molecular weights were calculated using a conventional calibration with polystyrene standards.

#### 2.3.3. Differential Scanning Calorimetry (DSC)

Differential Scanning Calorimetry measurements were performed using a TA Instruments Q2000 analyzer with a RC590 cooling system using a standard heat-cool-heat method. The temperature range was −50 °C to 200 °C. Both the heating and cooling rates were 10 °C/min in N_2_. The amount of sample was approximately 6 ± 0.1 mg. The samples were deposited in Tzero aluminum pans.

#### 2.3.4. Thermal Gravimetric Analysis (TGA)

The thermal stability of the polyesters was determined by TGA using TA Instruments Q5000 equipment under N_2_ atmosphere. The samples were placed in aluminum pans and heated from room temperature to 550 °C at a rate of 10 °C/min.

#### 2.3.5. Coatings Characterization

Coatings were applied at 18–20 microns directly onto a smooth polyester pre-primed steel substrate and cured for 40 s to reach a peak metal temperature of 224–232 °C. The physical testing methods carried out on the metal panels are described in Supplementary Material, Section 2 [55,56,57,58,59,60,61].

## 3. Results and Discussion

### 3.1. ^1^H NMR

The intention of the present ^1^H NMR analysis was to solely provide a general insight into the regions that identify the carbohydrate-derived polyesters. Furthermore, no quantitative analysis or precise definition of every signal was undertaken. The analysis by ^13^C NMR and 2D NMR could facilitate the study. The NMR spectra of the isosorbide monomer is included in Supplementary Material, Figure S1, whilst illustrative ^13^C NMR and 2D NMR spectra of a representative PPeFIS polyester are depicted in Figures S2 and S3.

**Figure 2:**
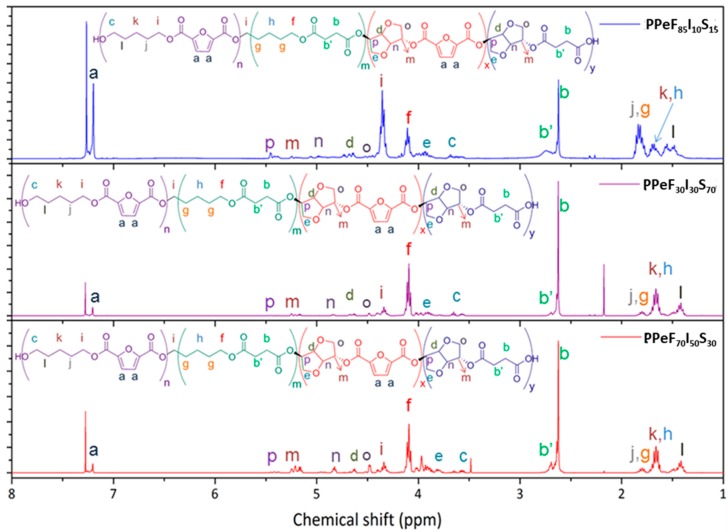
^1^H NMR spectra of PPeF_85_I_10_S_15_, PPeF_30_I_30_S_70_, and PPeF_70_I_50_S_30_. The mol % isosorbide increases from top to bottom.

The chemical shifts and assignments are summarized in Table 3. Figure 2 shows the NMR spectra for 1,5-pentanediol copolyesters PPeF_85_I_10_S_15_, PPeF_30_I_30_S_70_, and PPeF_70_I_50_S_30_, which contain 10, 30, and 50 mol % isosorbide, respectively. The identification of single peaks was a complex process since the incorporation of isosorbide could possibly facilitate the formation of cyclic structures and short chain oligomers, along with the probable presence of unreacted isosorbide (potentially between 3.5–4.5 ppm, Figure 2) at the end of the chains, leaving the secondary hydroxyl unreacted. The presence of impurities in the isosorbide monomer was a possibility as well (>98% purity), as it is one of the limitations of industrially-sourced isosorbide, along with residual monomer. 

The formation of the FDCA and succinic acid esters is confirmed by the shifts at 4.34 (*i*) and 4.09 (*f*), respectively, as the protons of the FDCA esters tend to shift to higher ppm values [29,30,31]. The assignment *a* (7.2 ppm) corresponds to the protons of the furan ring of FDCA. The signals between 3.8 ppm and 5.4 ppm (*e,d,m,n,o,p*) are attributed to the protons of isosorbide, which is in good agreement with previous literature of different isosorbide-based polyesters: 3.9–5.6 ppm [24], 3.7–5.2 ppm [17], 3.8–5.4 ppm [20], and 3.8–5.15 ppm [19]. The differences in shifts between peaks *p* (5.40–5.46 ppm) and *m* (5.21–5.25ppm) are the suggested result of the *endo* (*p*) and *exo* (*m*) stereochemistry of the two hydroxyl groups of isosorbide, as well as between peaks *d* (4.62–4.67 ppm) and *n* (4.84–4.97 ppm) [19]. Gioia et al. [24] identified the *endo* and *exo* OH groups at 5.2 ppm and 5.5 ppm, respectively. The broadening of the CH_2_-signal of succinic acid (*b* and *b’*, 2.62 and 2.69 ppm) was derived from the presence of two diols, 1,5-pentanediol and isosorbide, coupled with the *endo* and *exo* stereochemistry of isosorbide. A similar behavior was reported for polyesters conformed by 1,3-propanediol and isosorbide with succinic acid, where the succinic acid shifts were observable between 2.6 and 2.8 ppm [19]. Apparently, from PPeF_85_I_10_S_15_ down to PPeF_30_I_50_S_70_ with 50 mol % isosorbide, the characteristic peaks of 1,5-pentanediol (1.42–1.80 ppm) decrease as the isosorbide concentration increases [20], although a quantitative analysis should be performed to confirm the assumption.

### 3.2. GPC

**Figure 3:**
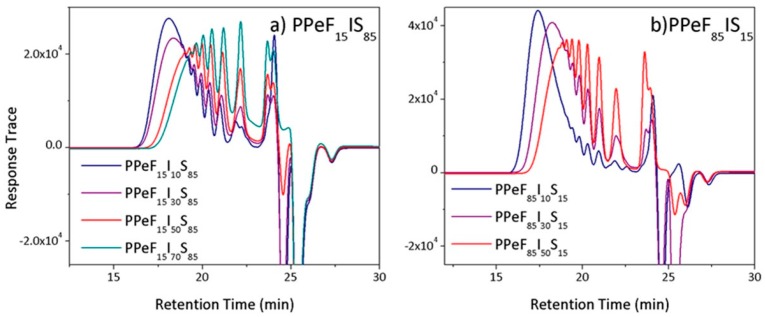
Gel permeation chromatography (GPC) chromatographs of (**a**) PPeF_15_IS_85_ and (**b**) PPeF_85_IS_15_.

M_n_, M_w,_ and Ð were measured by GPC. Table 1 and Table 2 show the results obtained for the isosorbide-based polyesters, whilst Figure 3 shows the chromatographs for the PPEF_15_IS_85_ and PPeF_85_IS_15_ families, respectively. 

**Figure 4:**
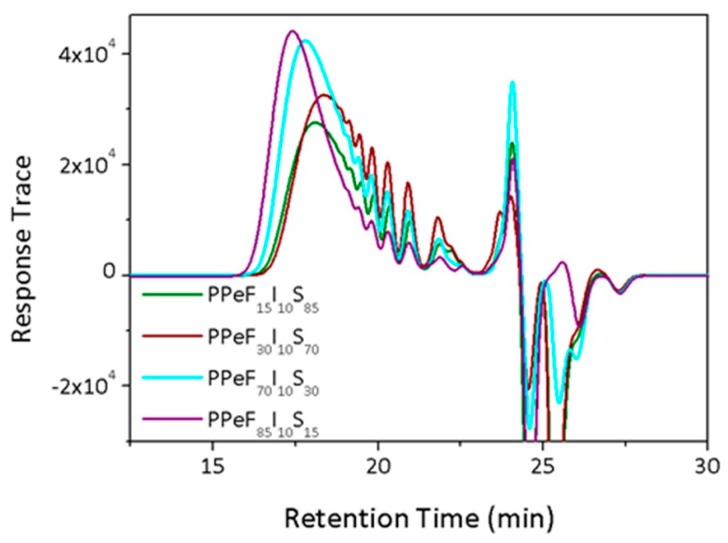
GPC chromatograms of polyesters PPeFIS with 10 mol % isosorbide.

The range of M_w_ for the polyesters was between 700 and 10200 Da, whereas M_n_ fell within 500 and 3100 Da, indicating the great influence that the addition of isosorbide imparts, which allows a great versatility within the properties of these biomass-derived polyesters. The results obtained suggest that in general, M_n_ and M_w_ decreased as the isosorbide content increased. The dispersity of PPeFIS fell within 1.5/2.4 which was close to the expected value of two for polyesters [62], whilst PPFIS was between 1.3–1.6, suggesting moderate polydisperse samples. The narrowness of the distributions with isosorbide concentrations from 10% to 30% (Figure 3 and Figure 4) were an indication of the strength and toughness of the polyesters. On the other hand, the narrow peaks at higher reaction times were an indication of the presence of short, oligomeric species, and potentially residual isosorbide, as previously seen in the NMR spectra. The highest M_n_ and M_w_ were achieved with the furan rich polyesters PPeF_85_IS_15_ and PPeF_70_IS_30_ and the trend is followed as the FDCA content decreased, although PPeF_70_IS_30_ had slightly higher molecular weight when the mol % isosorbide was between 30 and 50%. The incorporation of isosorbide was limited to 50 mol % for these two polyester families, as the mixture becomes extremely viscous and highly unprocessable above that concentration.

The molecular weight as a function of FDCA/succinic acid composition is depicted in Figure 4 for copolyesters bearing 10 mol % isosorbide. The chromatograms for 30 and 50 mol % isosorbide are available in Supplementary Material Figures S4 and S5.

The copolyesters PPeF_70_I_10_S_30_ and PPeF_85_I_10_S_15_ with 10 mol % isosorbide presented M_w_ of 3800 Da and 5400 Da, respectively and the dispersities of both compositions with different mol % isosorbide were above two. These values represent the highest molecular weights among the copolyesters of FDCA and succinic acid with isosorbide, whereas the lowest M_w_ figures correspond to PPeF_15_IS_85_ and PPeF_30_IS_70_ with either 60 or 70 mol % isosorbide (1000 Da). In the case of PPeF_15_IS_85_ and PPeF_30_IS_70_, dispersities of two and M_w_ above 2000 Da were obtained when the isosorbide content was limited to 30%.

The results for PPFIS resembled our findings with PPeFIS copolyesters. It was observed that M_n_ decreases as isosorbide content increases, for all compositions. The chromatograms of PPF_15_IS_85_ and PPF_30_IS_70_ are available in Supplementary Material Figure S6. Unfortunately, the results for PPF_70_IS_30_ and PPF_85_IS_15_ are not available since the samples were insoluble in THF.

The decrease in M_n_ as the isosorbide content increases has been reported in the literature [12,15,19,20,63]. One of the possible explanations was the decreased reactivity of the secondary OH groups when compared with the primary OH groups present in aliphatic linear diols, possibly corresponding to a lower acidic character [24], and most importantly, nucleophilicity. Nucleophilicity is affected by steric hindrance, since the bulkier a given nucleophile is, the slower the rate of its reactions and therefore the lower its nucleophilicity. In the case of isosorbide, although the nucleophilicity of the *endo* hydroxyl group is increased, the steric hindrance caused by hydrogen bonding makes the *exo* hydroxyl group more reactive [64]. Consequently, the difference in reactivity of the OH groups present in isosorbide was mainly due to their stereochemical nature—*endo* and *exo*—where the steric hindrance of the *endo* hydroxyl group is known to decrease the whole reactivity of the system [24]. The OH in *endo* position is more likely to form intra-molecular hydrogen bonding with the oxygen in main chains, while the other in *exo* position is more reactive in polycondensation reactions [65]. In addition, the *endo* hydroxyl is protected by the steric bulk of the rest of the molecule [66]. It might be worthwhile to do a kinetic study to explore different catalysts and reaction times and their influence on the final molecular weight of the polyesters. Wei et al. [20] showed that increasing the isosorbide content above 30% resulted in a significant decrease in the number average molecular weights of the polyesters. In fact, the M_n_ of the homopolymer poly(isosorbide sebacate) was lower than 3000 Da. Sadler et al. [63] synthesized unsaturated polyesters of phthalic anhydride, maleic anhydride, ethylene glycol, and isosorbide. The polyesters had isosorbide contents from 10 to 25 mol %, and the molecular weight decreased accordingly from 7000 to 3500 Da. In the same vein, Noordover et al. [19] reported M_n_ from 2000 to 3100 Da for polyesters of succinic acid and isosorbide, and M_n_ from 2700 to 4600 Da with the addition of 1,4-butanediol or neopentyl glycol as the second diol monomer. Our results suggest that the incorporation of more than 50 mol % isosorbide when processing via azeotropic distillation is considerably detrimental to the molecular weight of the polyesters, even though a catalyst is added to the reaction mixture. This could be due either to changes in stoichiometry, so more hydroxyl functionality will reduce the molecular weight, or due to the isosorbide reacting as a mono-functional monomer. If the isosorbide content is kept between 10 and 30 mol %, the molecular weights are in the desirable range for coatings (i.e., 2000 to 6000 Da) [5,19].

### 3.3. DSC

**Figure 5:**
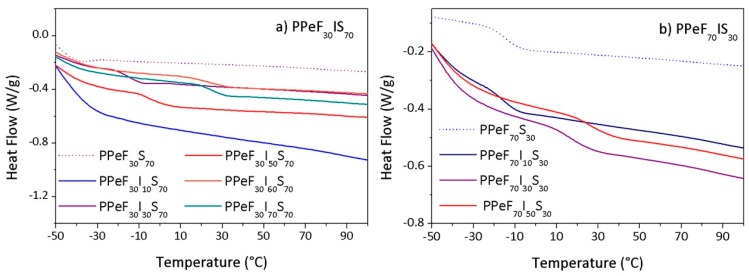
Second heating scan at 10 °C/min for polyesters (**a**) PPeF_30_IS_70_ including PPeF_30_S_70_ as the no-isosorbide reference and (**b**) PPeF_70_IS_30_ including PPeF_70_S_30_ as the no-isosorbide reference.

The DSC scans for PPeF_30_IS_70_ and PPeF_70_IS_30_ are shown in Figure 5, whilst the T_g_, melting temperatures (T_m_), and the melting enthalpies (ΔH_m_) are summarized in Table 4. The T_g_ of the parent resins with no isosorbide is included as reference. The isosorbide content and glass transition temperature kept a linear relationship as expected. In all the different compositions, the T_g_ increased as a function of the mol % isosorbide. Sadler et al. [63] demonstrated that adding as little as 10 mol % isosorbide to the reaction mixture in place of an equivalent amount of a linear diol resulted in a significant increase in T_g_. Moreover, adding 60 and 80 mol % isosorbide to resins based on succinic acid and neopentyl glycol achieved final glass transition temperatures from 30.5 °C and 47.1 °C, respectively [19]. 

Within the range of molecular weights of the resins and taking into account the diacid composition of the base polyester, a minimum content of 50 mol % isosorbide was needed in order to achieve glass transition temperatures above 0 °C and above room temperature. The copolyesters PPeF_70_IS_30_ and PPeF_85_IS_15_ exhibited the highest T_g_ among the 1,5-pentanediol polyesters, achieving values of 31 °C and 22 °C, respectively. Surprisingly, PPeF_70_I_50_S_30_ had higher T_g_ (31 °C) than PPeF_85_I_50_S_15_ (22 °C), with 50 mol % isosorbide, but when only 10 mol % was added, the difference in respect to the base polyester is either negligible (−11 °C for PPeF_70_I_10_S_30_ and −10 °C for PPeF_70_S_30_) or very little as per 85 mol % FDCA (4 °C, PPeF_85_I_10_S_15_ and 1 °C, PPeF_85_S_15_). Nonetheless, the slight difference might not be significant and could come down to the acid value that was processed.

**Figure 6:**
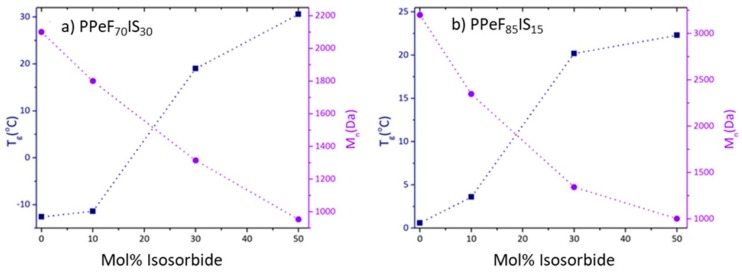
T_g_ and M_n_ as a function of mol % isosorbide for (**a**) PPeF_70_IS_30_ and (**b**) PPeF_85_IS_15_.

For the succinic acid rich polyesters PPeF_15_IS_85_ and PPeF_30_IS_70_, there is an abrupt increase in the glass transition temperature when the isosorbide content is 60% or above, going from values of approximately −40 °C up to 30 °C. This suggests that our library of polyester resins, based in three main monomers, would have different end properties just by tuning the desired content of isosorbide, expanding the potential applications of our renewable coatings. Figure 6 shows T_g_ and M_n_ as a function of isosorbide content for PPeF_70_IS_30_ and PPeF_85_IS_15_. The corresponding M_n_-T_g_-isosorbide relationships for PPeF_15_IS_85_ and PPeF_30_IS_70_ are depicted in Figures S10 and S11, Supplementary Material. It is observed how M_n_ decreases as the isosorbide content increases, as explained in the GPC section.

Nevertheless, some other factors, such as impurities, chemical conformation, degree of crystallinity, and molecular weight could be relevant for the analysis of thermal transitions. Consequently, further work could be focused on the particular effect of any of the above factors for selected polyesters, potentially those with closer molar masses or bearing the greatest industrial feasibility.

The glass transition temperatures of the PPFIS family, prepared with 1,3-propanediol, are slightly higher since they present lower chain flexibility than their 1,5-pentanediol counterparts. The results are available in Supplementary Material Table S3. The inclusion of 30 mol % isosorbide leads to an increase of T_g_ of about 30 °C (−45 °C to −14 °C) achieving a T_g_ at 6.2 °C with 70 mol % isosorbide. The copolyesters PPF_70_I_50_S_30_ and PPF_85_I_50_S_15_ with 50 mol % isosorbide exhibited the highest T_g_ among all the polyesters, achieving values of 29.2 and 53.2 °C, respectively. Representative DSC thermograms of PPFIS polyesters are available in Supplementary Material Figures S7 and S8.

Tailoring the different ratios of diacids and diols allowed to synthesize amorphous and semicrystalline polyesters. Regarding melting temperatures (T_m_) 1,5-pentanediol polyesters with FDCA contents of 30% and above showed melting endotherms from 77.3 °C to 154.7 °C, whereas for 1,3-propanediol the only observable Tm’s (97–131 °C) correspond to those containing 70 and 85 mol % FDCA. In the case of PPF_85_IS_15_ and PPeF_30_IS_70_, T_m_ decreased with an increase of the isosorbide content, a phenomenon that was previously observed in copolyesters of sebacic acid, 1,3-propanediol, and varying isosorbide content [12], whereas the other compositions exhibit the opposite trend. 

**Figure 7:**
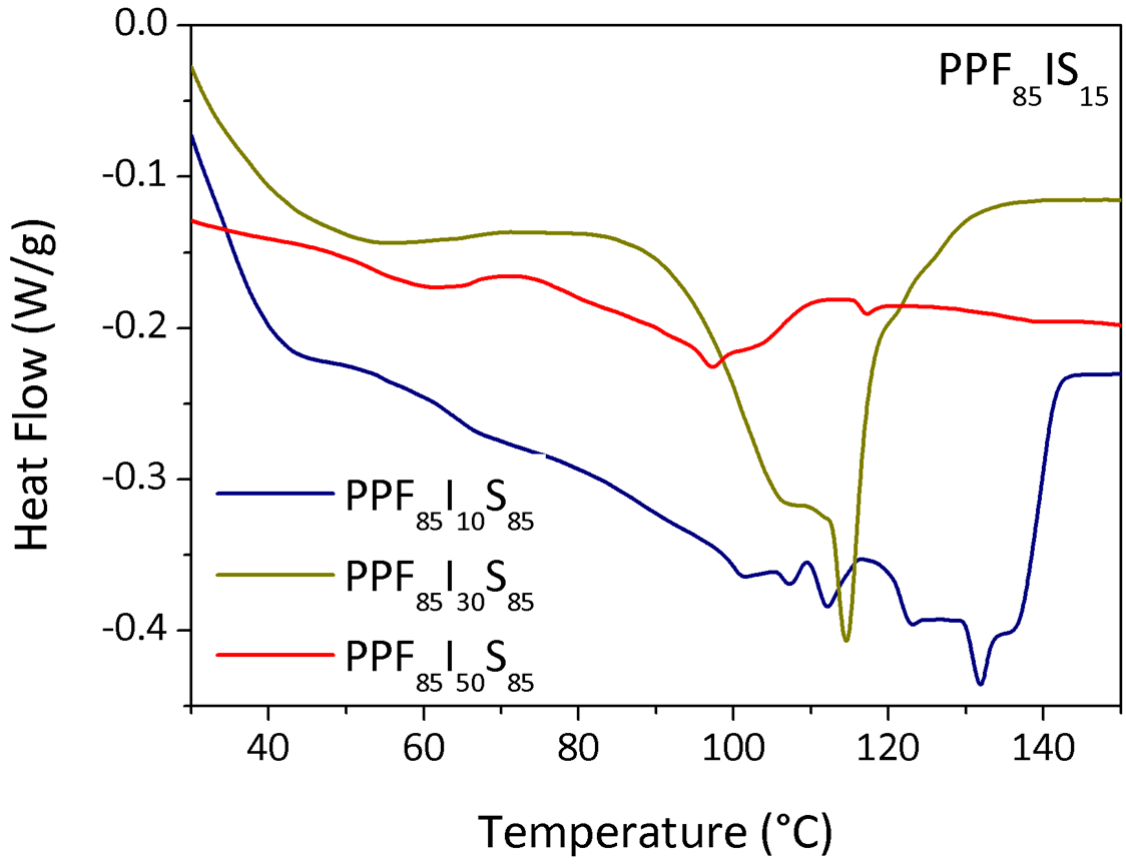
First heating scan at 10 °C/min for polyesters PPF_85_I_10_S_15_, PPF_85_I_30_S_15,_ and PPF_85_I_50_S_15_.

Particularly, polyesters PPF_70_IS_30_ and PPF_85_IS_15_ did not show a single melting peak in the DSC heating scan, as observed in Figure 7 for PPF_85_IS_15_ and Figure S9 for PPF_70_IS_30_. This behavior has been reported in the literature [12,67,68,69] for different copolyesters, and it is thought to be potentially the result of the existence of different crystal types in the same polymer sample [67], melting-recrystallization-remelting processes [68,70,71], or the presence of different molecular weight species [12]. It is necessary to confirm the assumptions regarding crystallinity features by performing an X-Ray Diffraction (XRD) analysis of the polyesters. There is a strong possibility that the polyesters are conformed by different oligomers with varying molecular weights, as the presence of isosorbide tends to form cyclic oligomeric structures [19,72]. Again, the confirmation of these structures requires further robust analysis, such as MALDI-TOF-MS, although this is outside our current scope.

Previous syntheses of isosorbide-derived polyesters have reported glass transition temperatures in the range presented herein or below. For instance, the T_g_’s of poly(isosorbide sebacate) and poly(isosorbide itaconate) were 6.4 °C (M_n_ = 19200 Da) and 34.5 °C (M_n_ = 8700 Da), respectively [17]. Wei et al. [20] synthesized polyesters of 1,10-decanediol, sebacic acid and isosorbide with different mol % isosorbide, namely, 43.9%, 66.2%, and 100%. The polyesters showed T_g_’s at −26 °C, −18 °C, and −5 °C, with M_w_ = 8900, 8699, and 2800 Da, respectively. The authors did not observe glass transitions for 5.3 or 16.7 mol % isosorbide, which they attributed to the fact that long chain aliphatic polyesters have a strong crystallization capacity and crystallize very fast. Terzopoulou and coworkers [22] showed that poly(isosorbide-2,5-furanoate) presented a Tg at 157 °C, whilst the Tg’s for copolyesters of FDCA, isosorbide, and 1,4-cyclohexane-dimethanol (CHDM) increased from 75.3 to 103.5 °C, with decreasing CHDM/isosorbide ratios from 95/5 to 60/40 [21].

### 3.4. TGA

**Figure 8:**
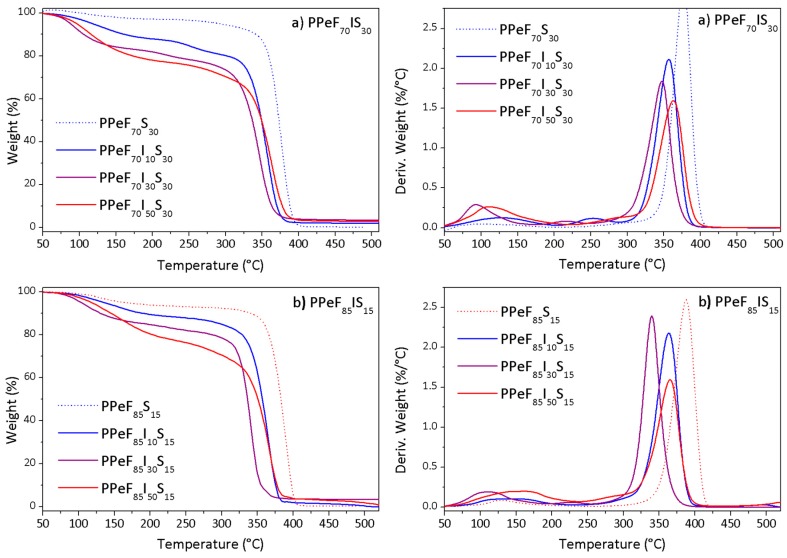
Weight % and derivative weight thermograms of polyesters (**a**) PPeF_70_IS_30_ and (**b**) PPeF_85_IS_15_ (N_2_ flow, 10 °C∙min^−1^).

Figure 8 shows the thermograms for the polyesters PPeF_70_IS_30_ and PPeF_85_IS_15_. Thermal gravimetric analysis indicates that the thermal stability ranges from 308 °C to 371 °C for the 1,5-pentanediol library. Expectedly, PPFIS polyesters showed a slightly higher and narrower T_d_ range between 362 and 372 °C, suggesting that the diol is a fundamental factor for thermal stability. Previous studies have shown that the thermal stability decreases with increasing methylene units. For example, the Bikiaris group [43] showed that poly(1,4-butylene furanoate) (PBF) was less thermally stable than the furanoates synthesized with shorter chain diols, such as ethylene glycol and 1,3-propanediol. The thermal decomposition temperatures (T_d_) for PPeFIS and PPFIS are summarized in Tables S4 and S5 in Supplementary Material, along with the TGA thermograms of PPeF_15_IS_85_ and PPeF_30_IS_70_, which correspond to Figure S12.

For both linear aliphatic diols, the incorporation of isosorbide to the main polymers resulted in a decrease of approximately 30–40 °C in the maximum T_d_ of the resins, which were close (~385 °C) or above 400 °C. Within each diacidcomposition, the results suggest that the PPeFIS polyesters with highest mol % isosorbide present the highest decomposition temperature. However, the same trend was not observed for PPFIS, where all the polyesters had similar T_d_ values. 

Specifically, for 1,3-propanediol, the thermogravimetric data suggests the presence of diverse species within the polymer matrix, as three different decomposition temperatures (T_d1_, T_d2_, and T_dmax_, Table S5) are observed. All the diacid compositions present the first decomposition temperature between 98.7 °C and 194 °C, followed by a second transition at 180–290 °C. The lowest T_d_’s appear to be prompted by isosorbide concentrations of above 50 mol %. The nature of these species has not been determined; however, previous research has shown that polyesters undergo decomposition mechanisms initiated by scission of an alkyl-oxygen bond, suggesting a random-chain scission [73]. Also, the decomposition of these polyesters is suggested to be dominated by cyclic or open chain oligomers with carboxylic-end groups. The formation of these cyclic oligomers is done through an intramolecular exchange reaction which happens below 300 °C [74] which could be the same process that took place in our polyesters. Cyclization of isosorbide-based polyesters has indeed been reported by other authors [15,19]. In the case of polyesters with low FDCA and rich isosorbide contents, data suggests the formation of more volatile products, which some authors have determined to arise from the secondary breakdown of end-groups, which follows the primary step of cyclic concerted decomposition [75].

### 3.5. Coatings

Table 5 and Table 6 summarize the results obtained. For succinic acid-rich polyesters, the chosen samples were those with higher isosorbide concentrations. A reference, petro-derived coating *R* has been included for comparison. Specifically, in the case of 1,3-propanediol resins, it was not possible to make paints with PPF_15_I_70_S_85_ and PPF_30_I_70_S_70_ (70 mol % isosorbide) as they were immiscible with the paint solvent system, which consisted of a 50:50 mixture of butanol and dibasic ester. In the case of PPF_70_I_50_S_30_, the resin was a brittle solid, which prevented solution in the same paint solvents. As similarly reported for some coating applications [14,19,24,63], the introduction of isosorbide into the synthesis of the polyester resins improved the thermomechanical properties of the resulting paints, compared to the parent polyesters. The no-isosorbide parent polyesters, PPeF1_5_S_85_ and PPeF_30_S_70_, had very low microhardness (11 N·m^−2^ and 12 N·m^−2^, respectively) and low T_g_ (−28 °C and −17 °C, respectively), but when 70 mol % isosorbide was added (PPeF_15_I_70_S_85_ and PPeF3_0_I_70_S_70_) the microhardness abruptly increased to 237 N·m^−2^ and 287 N·m^−2^, with T_g_ at 66 °C and 53 °C. The resins were not very flexible as T-Bend was 5T and 6T and both presented severe cracking after the Erichsen testing, which suggested that the concentration of FDCA should be increased. We previously reported the effect of the FDCA on the final polyester resin [2]. PPeF_70_I_30_S_30_ with an isosorbide concentration of 30 mol % and an increased FDCA concentration of 70% showed an improvement in flexibility (2.5T) respect to the reference resin (3T), T_g_ on specification at 34 °C and better impact resistance as it presented a slight cracking. Moreover, PPeF_70_I_50_S_30_ had above-specification Erichsen (No cracking), T_g_ (67 °C) and microhardness (270 N·m^−2^) than the reference resin. Likewise, comparing PPeF_70_I_50_S_30_ with its parent resin with no isosorbide, the performance was considerably improved, as the properties recorded for the latter were T_g_ = 6 °C, Microhardness = 20 N·m^−2^ and T-Bend no cracking of 1.5T. 

PPeF_85_IS_15_ resins again showed better microhardness and higher T_g_ than PPeF_85_S_15_, as the results were 21 N·m^−2^ and 15 °C, respectively. PPeF_85_I_50_S_15_ (Table 6), despite having the highest T_g_ (69 °C) and enhanced microhardness (299 N∙m^−2^) was slightly harder (Pencil Hardness 2H) and presents poor flexibility measured by T-Bend no cracking (5.5T). PPeF_85_I_30_S_15_ therefore presented the best overall properties (Table 6).

## 4. Conclusions

We successfully diversified and enhanced the properties of novel biomass-derived polyester coatings based on FDCA, succinic acid, and either 1,3-propanediol or 1,5-pentanediol by incorporating isosorbide into the polyesters’ backbone. Depending on the concentration of isosorbide, the molecular weight of the polyesters could be tuned from 700 to 10,200 Da. In general, the molecular weight decreased as the isosorbide content increased. This behavior could be attributed to the difference in reactivity of the OH groups present in isosorbide. The thermal results suggested that a minimum 50 mol % isosorbide is needed to achieve glass transition temperatures above 0 °C and above room temperature. The T_g_ could vary approximately 40 degrees by solely adjusting the molar isosorbide concentration. 

Paint testing results showed that the best isosorbide resins for coil coating applications were PPeF_85_I_30_S_15_ and PPeF_70_I_30_S_30_, along with PPF_85_I_30_S_15_. The consideration of bioderived 1,5-pentanediol as a main building block opens the possibility towards the development of flexible coatings but with better hardness than currently used diols, such as 1,6-hexanediol, enhanced by the presence of FDCA and isosorbide.

The inclusion of the carbohydrate-derived diol isosorbide to our coatings did promote better performance in terms of thermomechanical properties, and allowed for the tuning of fully biomass-derived resins that could easily replace the current petrochemical-derived ones in different applications, with controlled molecular weights and glass transition temperatures. Exterior durability testing for top coats would still be needed, but our bio-derived coatings could be used as backing coatings and interior finishes. Future work however needs to be done in terms of the variables effects on the T_g,_ the identification of the nature of the different oligomers or cyclic structures formed during the polymerization, as well as a kinetic study to optimize the reaction conditions to overcome the isosorbide’s low-reactive nature.

## Figures and Tables

**Table 1:** Synthesized biomass-derived polyesters with 1,3-propanediol and isosorbide (PPFIS)**.**

Polymer ^a^	Temperature, °C	Mol% FDCA	Mol% Isosorbide	R ^b^	M_n_, Da	M_w_, Da	Đ
PPF_15_I_30_S_85_			30		700	1100	1.6
PPF_15_I_60_S_85_	215	15	60	1.5	650	1000	1.5
PPF_15_I_70_S_85_			70		600	1000	1.5
PPF_30_I_30_S_70_			30		900	1500	1.7
PPF_30_I_60_S_70_	215	30	60	1.5	600	1000	1.6
PPF_30_I_70_S_70_			70		500	700	1.3
PPF_70_I_10_S_30_			10		- ^c^		
PPF_70_I_30_S_30_	215	70	30	1.5	- ^c^		
PPF_70_I_50_S_30_			50		-		
PPF_85_I_10_S_15_			10		- ^c^		
PPF_85_I_30_S_15_	215	85	30	1.5	-		
PPF_85_I_30_S_15_			50		-		

^a^ Catalyst: 0.02 mol % SnCl_2_/mol diacids, ^b^ Molar ratio diols:diacids. ^c^ Polymer insoluble in tetrahydrofuran.

**Table 2:** Synthesized biomass-derived polyesters with 1,5-pentanediol and isosorbide (PPeFIS).

Polymer ^a^	Temperature, C	mol % FDCA	mol % Isosorbide	r ^b^	M_n_, Da	M_w_, Da	Đ
PPeF_15_I_10_S_85_			10		1500	3100	2.1
PPeF_15_I_30_S_85_			30		1200	2500	2.2
PPeF_15_I_50_S_85_	215	15	50	1.5	800	1500	1.8
PPeF_15_I_60_S_85_			60		500	1100	2.1
PPeF_15_I_70_S_85_			70		700	1100	1.7
PPeF_30_I_10_S_70_			10		1300	2700	2.0
PPeF_30_I_30_S_70_			30		1100	2800	2.5
PPeF_30_I_50_S_70_	215	30	50	1.5	900	1600	1.9
PPeF_30_I_60_S_70_			60		500	700	1.5
PPeF_30_I_70_S_70_			70		600	1000	1.6
PPeF_70_I_10_S_30_			10		1800	3800	2.1
PPeF_70_I_30_S_30_	215	70	30	1.5	1300	3200	2.4
PPeF_70_I_50_S_30_			50		1000	1900	2.0
PPeF_85_I_10_S_15_			10		2300	5400	2.3
PPeF_85_I_30_S_15_	215	85	30	1.5	1300	2800	2.1
PPeF_85_I_30_S_15_			50		1000	1800	1.8

^a^ Catalyst: 0.02 mol % SnCl_2_/mol diacids, ^b^ Molar ratio diols:diacids.

**Table 3:** Assignment of chemical shifts of PPeFIS and integrations for PPeF_85_I_10_S_15._

Polyester	Assignment of Chemical Shifts (CDCl_3_, δ/ppm)						
PPeFIS	a	b,b’	c	d	e	f	g,j
	7.20	2.62, 2.69	3.68	4.62–4.67	3.80–3.97	4.09	1.80
	h,k	i	l	m	n	o	p
	1.66	4.34	1.42–1.48	5.21–5.25	4.84–4.97	4.48	5.40–5.46
Integrations for PPeF_85_I_10_S_15_							
	a	b,b’	c	d	e	f	g,j
	2.03	2.00, 1.13	0.47	0.53	1.17	2.24	4.05
	h,k	i	l	m	n	o	p
	1.97	4.57	2.95	0.14	0.21	0.21	0.47

**Table 4:** Thermal transitions of PPeFIS polyesters measured by differential scanning calorimetry (DSC).

Polyester	Mol% Isosorbide	M_w_, Da	T_g_, °C	T_m_, °C	ΔH_m_, J/g
PPeF_15_S_85_	0	2700	−46	-	-
PPeF_15_I_10_S_85_	10	3100	−43	-	-
PPeF_15_I_30_S_85_	30	2500	−26	-	-
PPeF_15_I_50_S_85_	50	1500	−12	-	-
PPeF_15_I_60_S_85_	60	1100	7	-	-
PPeF_15_I_70_S_85_	70	1100	35	117	2.6
PPeF_30_S_70_	0	2800	−39	-	-
PPeF_30_I_10_S_70_	10	2600	−39	-	-
PPeF_30_I_30_S_70_	30	2800	−12	155	3.5
PPeF_30_I_50_S_70_	50	1600	0.7	139	1.3
PPeF_30_I_60_S_70_	60	700	23	105	0.1
PPeF_30_I_70_S_70_	70	1000	24	77	-
PPeF_70_S_30_	0	5900	−10	-	-
PPeF_70_I_10_S_30_	10	3800	−11	-	-
PPeF_70_I_30_S_30_	30	3200	19	94	0.4
PPeF_70_I_50_S_30_	50	1900	31	105	1.2
PPeF_85_S_15_	0	4100	1	50	-
PPeF_85_I_10_S_15_	10	5400	4	119	0.1
PPeF_85_I_30_S_15_	30	2800	20	101	1.0
PPeF_85_I_50_S_15_	50	1800	22	139	1.4

**Table 5:** Physical test results on white paints based on polyesters PPeF_15_IS_85_-PPeF_70_IS_30_ and the standard reference resin R.

Test	Standard	R	PPeF_15_I_70_S_85_	PPeF_30_I_70_S_70_	PPeF_70_I_30_S_30_	PPeF_70_I_50_S_30_
Pencil hardness	EN13523-4	H	2H	3H	H	H
Gloss top coat	13523-2	35	39	35	40	39
Reverse impact 80” lb	13523-5	Moderate cracking	Moderate cracking	Moderate cracking	Slight cracking	Moderate cracking
Erichsen 7.5 mm	13523-6	Moderate cracking	Severe cracking	Severe cracking	Slight cracking	No cracking
T-bend (no tape pick off)	13523-7	0.5T	1T	1.5T	0T	1T
T-Bend no cracking	13523-7	3T	5T	6T	2.5T	5T
MEK^a^ rubs primer	BSSP 3.522.11	110	110	110	110	110
T_g_, °C (onset/midpoint)		28/35	53/66	51/53	27/34	53/67
Microhardness, N∙m^−2^		216	237	287	190	270

^a^ Methyl ethyl ketone.

**Table 6:** Physical test results on white paints based on polyesters PPeF_85_I_10_S_15_, PPeF_85_I_30_S_15_, PPeF_85_I_50_S_15_, PPF_85_I_30_S_15_ and the standard reference resin R.

Test	Standard	R	PPeF_85_I_10_S_15_	PPeF_85_I_30_S_15_	PPeF_85_I_50_S_15_	PPF_85_I_30_S_15_
Pencil hardness	EN13523-4	H	H	F	2H	H
Gloss top coat	13523-2	35	40	43	38	39
Reverse impact 80” lb	13523-5	Moderate cracking	No cracking	Moderate cracking	Severe cracking	Slight cracking
Erichsen 7.5 mm	13523-6	Moderate cracking	No cracking	No cracking	Severe cracking	Slight cracking
T-bend (no tape pick off)	13523-7	0.5T	1.5T	1.5T	1T	1.5T
T-Bend no cracking	13523-7	3T	1T	2T	5.5T	2.5T
MEK rubs primer	BSSP 3.522.11	110	110	110	110	110
T_g_, °C (onset/midpoint)		28/35	26/32	34/42	58/69	29/34
Microhardness, N∙m^−2^		216	174	287	299	169

## Supplementary Materials

The following are available online at http://www.mdpi.com/2073-4360/10/6/600/s1, Figure S1: ^1^H NMR of isosorbide monomer, Table S1: Monomer Charge for PPFIS Polyesters, Table S2: Monomer Charge for PPeFIS polyesters, Figure S2: ^13^C NMR of PPeF_15_I_50_S_85_, Figure S3: HSQC of PPeF_15_I_50_S_85_, Figure S4: GPC chromatogram of polyesters PPeFIS with 30 mol % isosorbide, Figure S5: GPC chromatogram of polyesters PPeFIS with 50 mol % isosorbide, Figure S6: GPC chromatogram of polyesters PPF_15_IS_85_ and PPF_30_IS_70_, Table S3: Thermal transitions of PPFIS measured by DSC, Figure S7: DSC thermogram of PPeF_15_IS_85_, Figure S8: DSC thermogram of PPF_30_IS_70_, Figure S9: First heating scan at 10 °C/min for polyesters PPF_70_IS_30_, Figure S10: T_g_-M_n_-mol % isosorbide relationship for PPeF1_5_IS_85_, Figure S11: T_g_-M_n_-mol % isosorbide relationship for PPeF1_30_IS_70_, Table S4: Characteristic decomposition temperatures T_d1_, T_dmax_ and weight loss% of PPeFIS, Table S5: Characteristic decomposition temperatures T_d1_, T_d2_ and T_dmax_ and weight loss% of PPFIS, Figure S12: TGA thermograms for PPeF_15_IS_85_ and PPeF_30_IS_70_.
