# Supplementary material for: Synthesis and Characterization of Renewable Polyester Coil Coatings from Biomass-Derived Isosorbide, FDCA, 1,5-Pentanediol, Succinic Acid, and 1,3-Propanediol
Source: Polymers (Basel). 2018 May 29;10(6):600. doi: 10.3390/polym10060600 (PMC6404048; doi:10.3390/polym10060600)
Supplement: Supplementary file 1 [file polymers-10-00600-s001.pdf]

Supplementary Materials

Synthesis and characterization of renewable polyester coil coatings from biomass-derived isosorbide, FDCA, 1,5-pentanediol and 1,3-propanediol

Mónica Lomelí-Rodríguez[1], José Raúl Corpas-Martínez[1], Susan Willis[2], Robert Mulholland[2] and Jose Antonio Lopez-Sanchez[1*]

[1]Stephenson Institute for Renewable Energy, Department of Chemistry, University of Liverpool, Crown Street, Liverpool, L69 7ZD- UK.

[2] Industrial Coatings Ltd, Goodlass Road, Speke, Liverpool, L24 9HJ, UK

1. Experimental details and purification method

IS: Isosorbide; SA: succinic acid; FDCA: 2,5-furan dicarboxylic acid, PDO: 1,3-propanediol; PTO: 1,5-pentanediol.

Table S1. Monomer Charge for PPFIS polyesters

	**Mol%**	**Mol**				**Mass, g**			
**Polyester**	**IS**	**IS**	**SA**	**FDCA**	**PDO**	**IS**	**SA**	**FDCA**	**PDO**
**$PPF_{15}I_{30}S_{85}$**	30	0.32	0.60	0.11	0.74	46.03	70.26	16.39	55.93
**$PPF_{15}I_{60}S_{85}$**	60	0.6	0.57	0.1	0.4	87.68	66.92	15.61	30.44
**$PPF_{15}I_{70}S_{85}$**	70	0.69	0.56	0.1	0.3	102	66.92	15.61	22.83
**$PPF_{30}I_{30}S_{70}$**	30	0.28	0.44	0.19	0.69	41.65	52.35	29.66	52.55
**$PPF_{30}I_{60}S_{70}$**	60	0.6	0.37	0.16	0.40	87.68	44.09	24.97	30.44
**$PPF_{30}I_{70}S_{70}$**	70	0.7	0.47	0.2	0.3	102.3	55.11	31.22	22.8

**PPF70I10S30**	10	0.19	0.38	0.89	1.71	27.76	44.87	138.4	130.11
**PPF70I30S30**	30	0.42	0.28	0.65	0.98	61.37	33.07	101.98	74.57
**PPF70I50S30**	50	0.87	0.35	0.82	0.88	127.87	41.33	127.47	66.58
**PPF85I10S15**	10	0.19	0.19	1.07	1.71	27.76	22.44	168.0	130.11
**PPF85I30S15**	30	0.55	0.19	1.05	1.3	81.1	21.85	163.63	98.54
**PPF85I50S15**	50	0.8	0.16	0.91	0.8	116.91	18.89	141.52	60.87

Table S2. Monomer Charge for PPeFIS polyesters

	**Mol%**	**Mol**				**Mass, g**			
**Polyester**	**IS**	**IS**	**SA**	**FDCA**	**PTO**	**IS**	**SA**	**FDCA**	**PTO**
**PPeF15I10S85**	10	0.08	0.56	0.1	0.77	12.42	65.63	15.31	79.67
**PPeF15I30S85**	30	0.25	0.56	0.1	0.6	37.27	65.63	15.31	61.96
**PPeF15I50S85**	50	0.45	0.59	0.1	0.45	65.76	69.49	16.21	46.86
**PPeF15I60S85**	60	0.15	0.16	0.03	0.10	21.92	19.3	4.5	10.41
**PPeF15I70S85**	70	0.59	0.56	0.1	0.26	86.95	65.63	15.31	26.56
**PPeF30I10S70**	10	0.08	0.46	0.2	0.77	12.42	54.05	30.62	79.67
**PPeF30I30S70**	30	0.25	0.45	0.19	0.59	37.26	54.05	30.62	61.96
**PPeF30I50S70**	50	0.42	0.46	0.2	0.43	62.1	54.05	30.62	44.26
**PPeF30I60S70**	60	0.15	0.13	0.06	0.10	21.92	15.9	9.01	10.41
**PPeF30I70S70**	70	0.59	0.46	0.2	0.26	86.95	54.05	30.62	26.56
**PPeF70I10S30**	10	0.15	0.35	0.81	1.35	21.92	40.88	126.0	140.59

**$PPeF_{70}I_{30}S_{30}$**	30	0.51	0.39	0.91	1.19	74.53	46.33	142.88	123.93
**$PPeF_{70}I_{50}S_{30}$**	50	0.75	0.35	0.81	0.75	109.6	40.88	126.0	78.11
**$PPeF_{85}I_{10}S_{15}$**	10	0.15	0.17	0.98	1.35	21.92	20.44	153.09	140.6
**$PPeF_{85}I_{30}S_{15}$**	30	0.45	0.17	0.98	1.05	65.73	20.44	153.09	109.35
**$PPeF_{85}I_{50}S_{15}$**	50	0.75	0.17	0.98	0.75	109.6	20.44	153.09	78.11

1.2 Purification method for $PPeF_{15}IS_{85}$, $PPeF_{30}IS_{70}$, $PPF_{15}IS_{85}$ and $PPF_{30}IS_{70}$

1. Fill the beaker up to a 10% of its total height with hexane, stir magnetically and ensure that the solvent does not evaporate and that the resin is completely dissolved in it.
2. Once this mixing is achieved, top up with methanol. To ensure an efficient contact of the hexane-dissolved resin phase with methanol a gentle stirring may be applied. After this, cover with Parafilm and allow settling and cooling down in ice. This stirring shall never create a suspension. Once there are to different phases observed (1-2 h), remove the largest amount of the above phase (disposing it in the solvent container). Remove the rest of this phase with a syringe, drawing out the minimum amount possible of the denser phase.
3. Vacuum-dry overnight in the oven at 50ºC.

For $PPeF_{85}IS_{15}$, $PPeF_{70}IS_{30}$, $PPF_{85}IS_{15}$ and $PPF_{70}IS_{30}$, the same procedure is followed, but chloroform is used instead of hexane as the solvent in step 1.

2. Paint testing characterization methods

The following physical testing was carried out on the metal panels:

- **Erichsen** EN 13523-6 (2002) Part 6 or ASTM D1474

Indentation hardness measurements have proven to be useful in rating coatings on rigid substrates for their resistance to mechanical abuse, such as that produced by blows and scratching [32].

- **Pencil Hardness** EN 13523-4 (2001): Part 4 or ASTM 3363-00

This part describes the procedure to assess the relative hardness of an organic coating on a metal substrate, by means of pencils of known hardness. The hardest lead which does not scratch the coating for a minimum of 3 mm length determines the degree of hardness [33].

- **Gloss (60º)** EN 13523-2 (2001) or ASTM D523-89(1999)

This test method covers the measurement of the specular gloss of nonmetallic specimens for glossmeter geometries of 60, 20, and 85°. Gloss is associated with the capacity of a surface to reflect more light in some directions than in others. The directions associated with specular reflection normally have the highest reflectance [34].

- **MEK Resistance** ECCA - T11 (1999) or ASTM D5402-93(1999).

This practice describes a solvent rub technique for assessing the solvent resistance of an organic coating that chemically changes during the curing process. Coatings that chemically change during the curing process, such as polyesters, become more resistance to solvents as they cure [35].

- **Micro-indentometry** ISO 14577-1.

Hardness has typically been defined as the resistance of a material to permanent penetration by another harder material [36].

- **Glass transition temperature** ASTM E1356-98.

This test method involves continuously monitoring the difference in heat flow into, or temperature between, a reference material and a test material when they are heated or cooled at a controlled rate through the glass transition region of the test material [37].

- **T – Bend Flexibility** No Crack / No Removal: EN 13523-7 (2001) Part 7 or ASTM D4145.

This test method describes a procedure for determining the flexibility and adhesion of coatings on metallic substrates that are deformed by bending when the sheet is fabricated into building panels or other products [38].

3. ^{1}H NMR of isosorbide

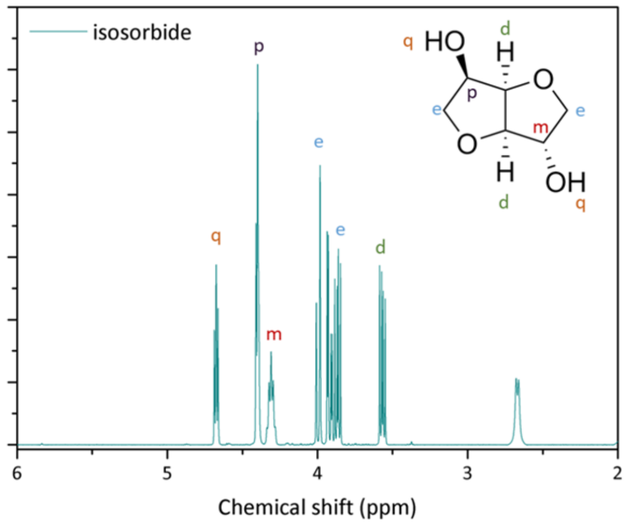

Figure S1. ^{1}H NMR spectra of isosorbide monomer.

^{13}C NMR, 2D NMR spectra of PPeFIS

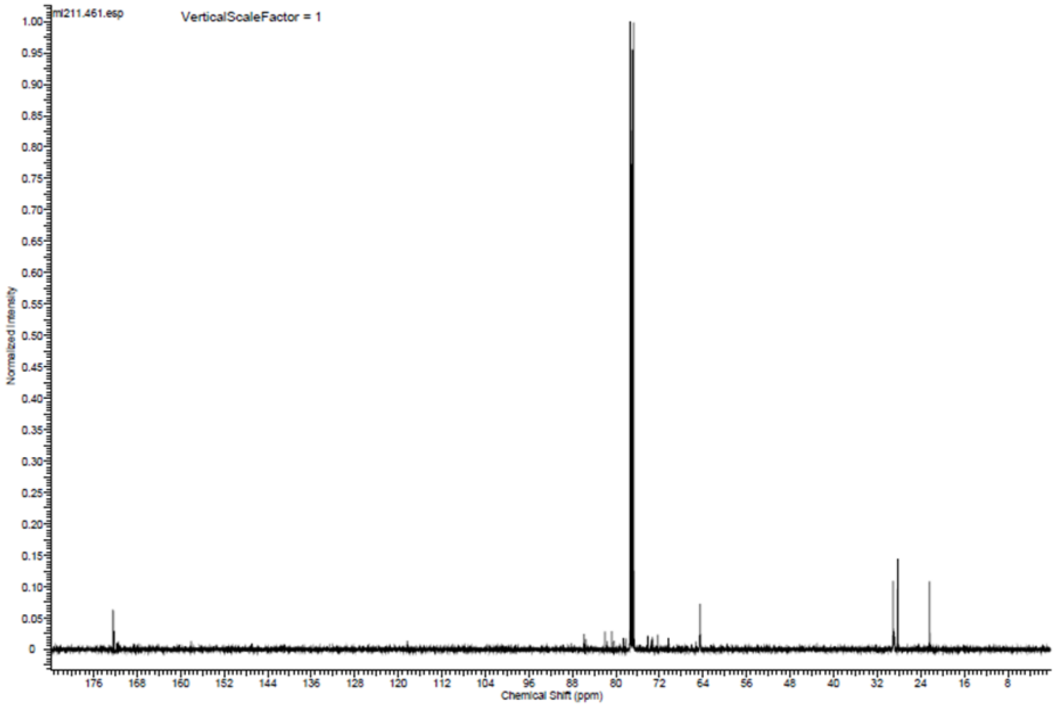

Figure S2. ^{13}C NMR of $PPeF_{15}I_{50}S_{85}$.

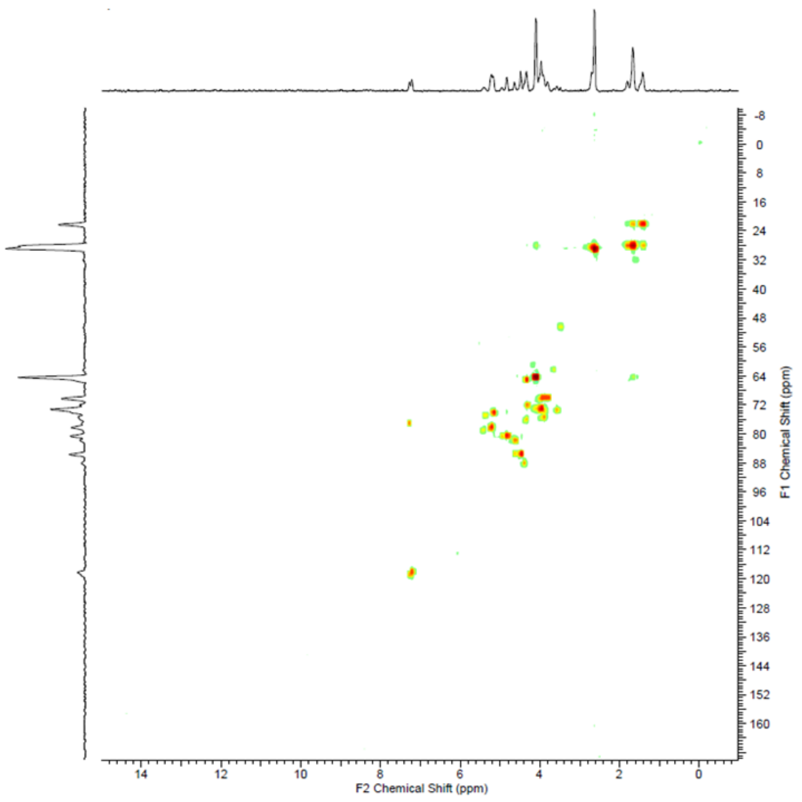

Figure S3. HSQC of $PPeF_{15}I_{50}S_{85}$.

4. GPC chromatographs

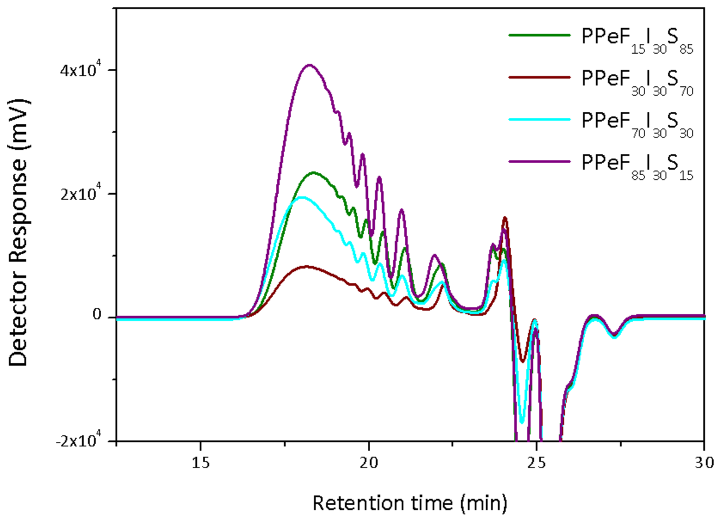

Figure S4. GPC chromatogram of polyesters PPeFIS with 30 mol% isosorbide.

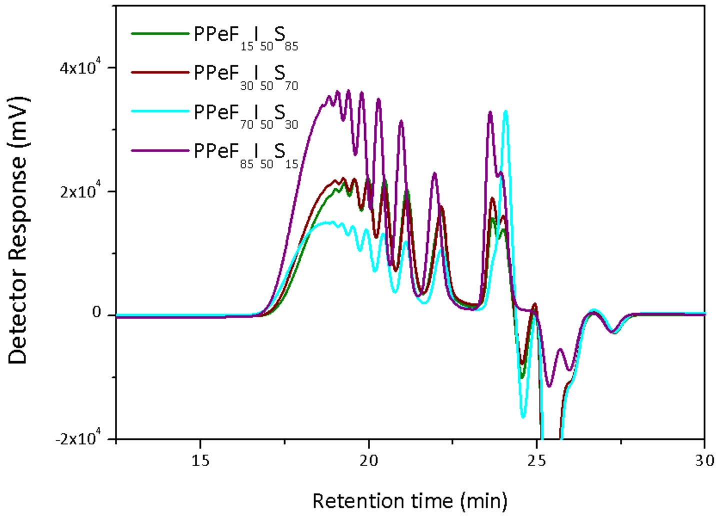

Figure S5. GPC chromatogram of polyesters PPeFIS with 50 mol% isosorbide.

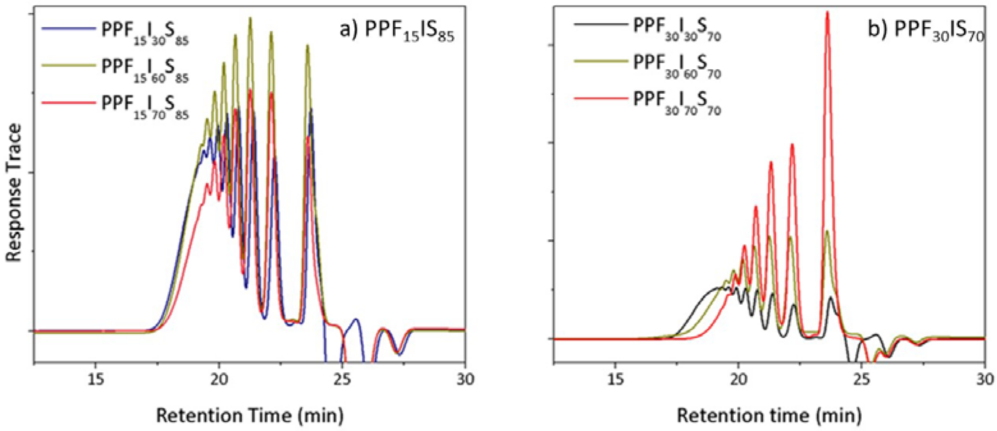

Figure S6. GPC chromatogram of polyesters a) $PPF_{15}IS_{85}$ and b) $PPF_{30}IS_{70}$

5. DSC

Table S3. Thermal transitions of PPFIS measured by DSC

Code	Mol% Isosorbide	M_w, Da	T_g, °C	T_m, °C
$PPF_{15}S_{85}$	0	1200	-45	-

$PPF_{15}I_{30}S_{85}$	30	1100	-14	-
$PPF_{15}I_{60}S_{85}$	60	1000	0	-
$PPF_{15}I_{70}S_{85}$	70	1000	6	-
$PPF_{30}S_{70}$	0	1200	-39	-
$PPF_{30}I_{30}S_{70}$	30	1500	4	-
$PPF_{30}I_{60}S_{70}$	60	1000	10	-
$PPF_{30}I_{70}S_{70}$	70	700	-36	-
$PPF_{70}S_{30}$	0	1700	2	111.7
$PPF_{70}I_{10}S_{30}$	10	-	-6	97.0
$PPF_{70}I_{30}S_{30}$	30	-	8	98.0
$PPF_{70}I_{50}S_{30}$	50	-	29	113.7
$PPF_{85}S_{15}$	0	1400	10	133.6
$PPF_{85}I_{10}S_{15}$	10	-	2	131.6
$PPF_{85}I_{30}S_{15}$	30	-	20	114.6
$PPF_{85}I_{50}S_{15}$	50	-	53	97.1

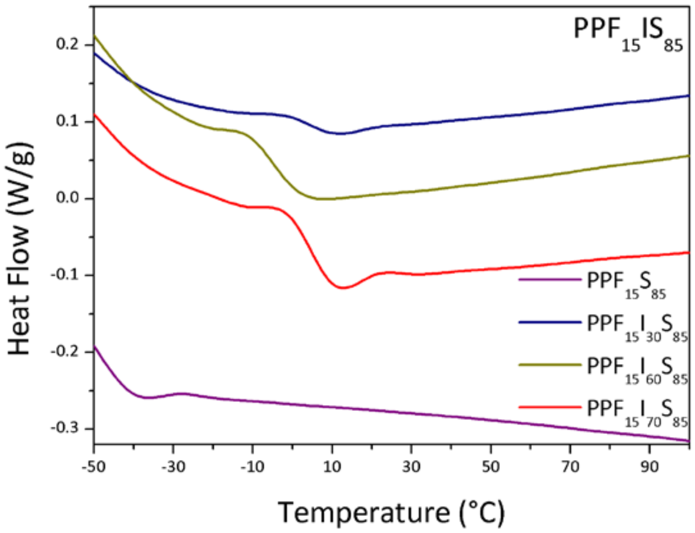

Figure S7. DSC thermogram of $PPF_{15}IS_{85}$.

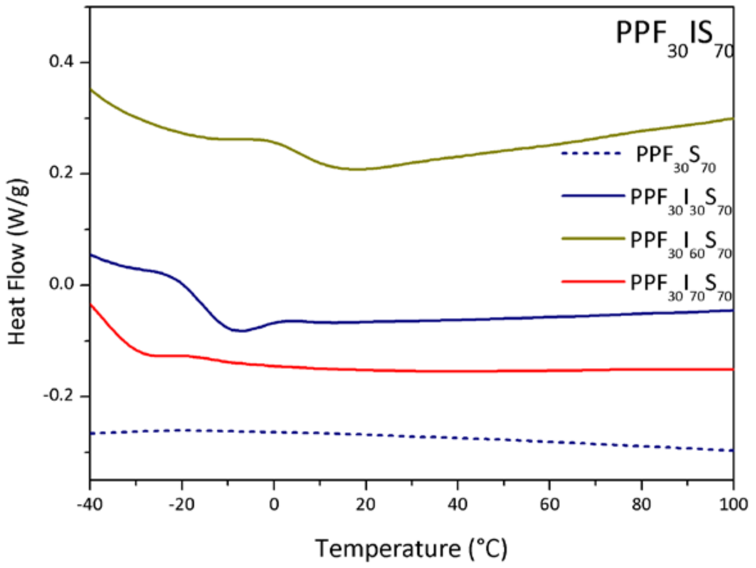

Figure S8. DSC thermogram of $PPF_{30}IS_{70}$.

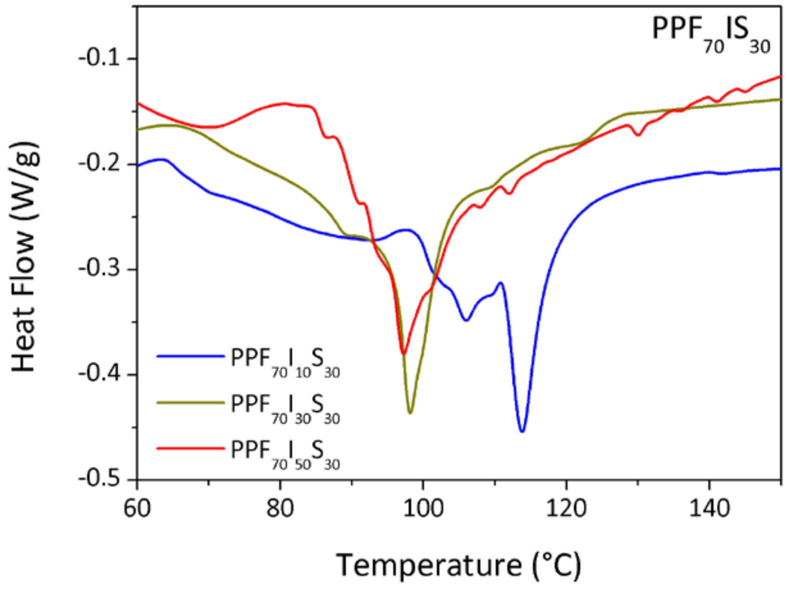

Figure S9. First heating scan at 10 °C/min for polyesters $PPF_{70}IS_{30}$.

T_g-M_n-mol% isosorbide graphs

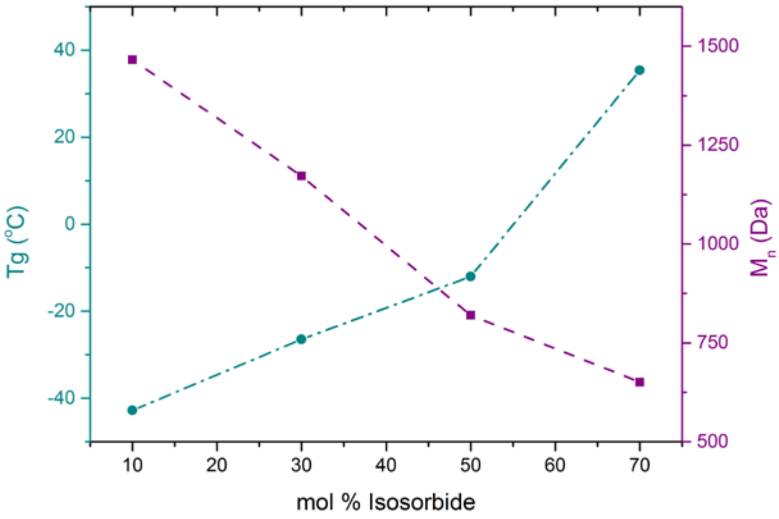

Figure S10. T_g-M_n- mol% isosorbide relationship for $PPeF_{15}IS_{85}$.

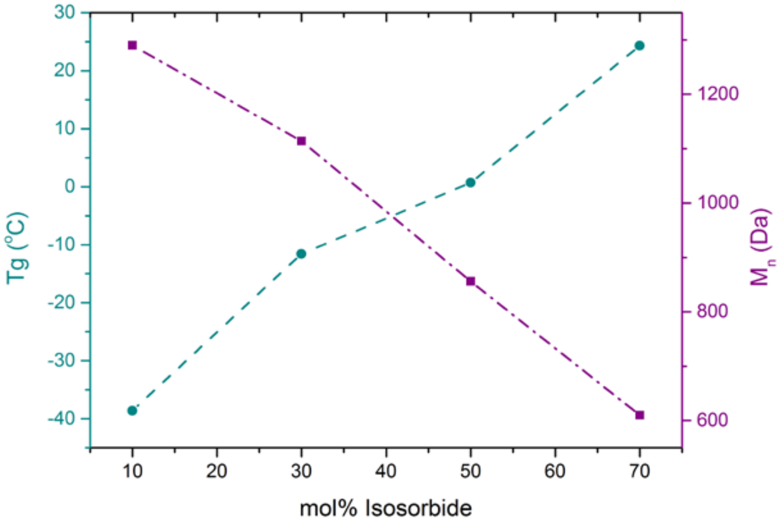

Figure S11. T_g-M_n- mol% isosorbide relationship for $PPeF_{30}IS_{70}$.

6. TGA

Table S4. Characteristic decomposition temperatures T_{d1}, T_{dmax} and weight loss % of PPeFIS

Polyester	Mol% Isosorbide	T_{d1}, °C	T_{dmax}, °C	Weight loss % (T_{dmax})
$PPeF_{15}S_{85}$	0	298.1	399.6	99.9
$PPeF_{15}I_{10}S_{85}$	10	277.3	365.8	98.2

PPeF$_{15}$I$_{30}$S$_{85}$	30	-	370.2	97.9
PPeF$_{15}$I$_{50}$S$_{85}$	50	228.5	368.3	98.4
PPeF$_{15}$I$_{60}$S$_{85}$	60	273.8	373.8	98.8
PPeF$_{15}$I$_{70}$S$_{85}$	70	214.3	371.2	96.8
PPeF$_{30}$S$_{70}$	0	140.9	403.4	99.8
PPeF$_{30}$I$_{10}$S$_{70}$	10	284.0	361.6	97.3
PPeF$_{30}$I$_{30}$S$_{70}$	30	125.4	362.4	97.2
PPeF$_{30}$I$_{50}$S$_{70}$	50	127.4	366.7	97.6
PPeF$_{30}$I$_{60}$S$_{70}$	60	207.0	368.8	93.9
PPeF$_{30}$I$_{70}$S$_{70}$	70	237.7	369.8	96.9
PPeF$_{70}$S$_{30}$	0	-	389.1	97.4
PPeF$_{70}$I$_{10}$S$_{30}$	10	252.9	357.7	97.8
PPeF$_{70}$I$_{30}$S$_{30}$	30	89.9	348.1	96.6
PPeF$_{70}$I$_{50}$S$_{30}$	50	110.4	340.9	97.1
PPeF$_{85}$S$_{15}$	0	104.4	385.3	91.1
PPeF$_{85}$I$_{10}$S$_{15}$	10	-	364.3	99.8
PPeF$_{85}$I$_{30}$S$_{15}$	30	114.9	340.9	96.4
PPeF$_{85}$I$_{50}$S$_{15}$	50	158.7	366.1	99.8

Table S5. Characteristic decomposition temperatures T_{d1}, T_{d2}, T_{dmax} and weight loss % of PPFIS

**Polyester**	**Mol% Isosorbide**	**T_{d1}, °C**	**T_{d2}, °C**	**T_{dmax}, °C**	**Weight loss % (T_{dmax})**
PPF$_{15}$S$_{85}$	0	298.6	-	401.0	99.2
PPF$_{15}$I$_{30}$S$_{85}$	30	181.9	281.1	370.5	98.3
PPF$_{15}$I$_{50}$S$_{85}$	60	176.9	290.9	371.1	97.1
PPF$_{15}$I$_{70}$S$_{85}$	70	174.1	278.2	368.7	98.3
PPF$_{30}$S$_{70}$	0	299.5	-	395.9	98.5
PPF$_{30}$I$_{30}$S$_{70}$	30	163.5	288.6	367.2	97.1
PPF$_{30}$I$_{60}$S$_{70}$	60	122.5	180.3	372.0	97.7
PPF$_{30}$I$_{70}$S$_{70}$	70	194.0	253.7	368.8	98.7

PPF$_{70}$S$_{30}$	0	299.0	-	395.9	98.5
PPF$_{70}$I$_{10}$S$_{30}$	10	280.3	-	367.2	97.1
PPF$_{70}$I$_{30}$S$_{30}$	30	266.1	-	372.0	97.7
PPF$_{70}$I$_{50}$S$_{30}$	50	98.7	-	368.8	98.7
PPF$_{85}$S$_{15}$	0	291.8	-	393.8	95.0
PPF$_{85}$I$_{10}$S$_{15}$	10	273.8	-	371.5	97.5
PPF$_{85}$I$_{30}$S$_{15}$	30	143.4	277.4	363.0	99.4
PPF$_{85}$I$_{50}$S$_{15}$	50	113.8	290.0	361.8	93.1

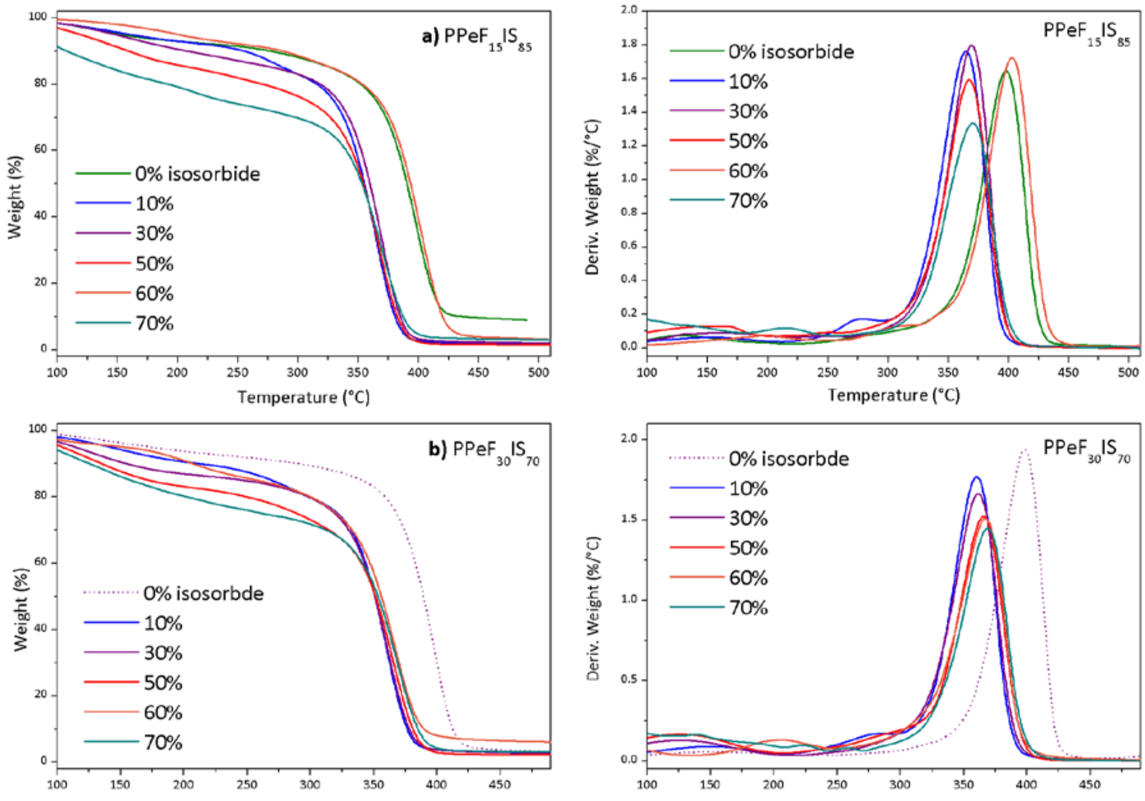

Figure S12. TGA thermograms for a) PPeF$_{15}$IS$_{85}$ and b) PPeF$_{30}$IS$_{70}$.
